# CircREOS suppresses lipid synthesis and osteosarcoma progression through inhibiting HuR-mediated MYC activation

**DOI:** 10.7150/jca.83106

**Published:** 2023-04-02

**Authors:** Weilai Tong, Shijiang Wang, Cheng He, Anan Li, Jiangbo Nie, Wei Zuo, Feng Yang, Zhili Liu

**Affiliations:** 1 Medical Innovation Center, The First Affiliated Hospital of Nanchang University, Jiangxi Medical College of Nanchang University, Nanchang, 330006, People's Republic of China.; 2 Department of Orthopedic Surgery, The First Affiliated Hospital of Nanchang University, Nanchang, 330006, People's Republic of China.; 3 Department of Orthopedics, the 908th Hospital of Joint Logistics Support Forces of Chinese PLA, Nanchang, 330006, People's Republic of China.

**Keywords:** MYC, Osteosarcoma, circREOS, HuR, lipid metabolism

## Abstract

MYC proto-oncogene (MYC) is a transcription factor among the most commonly activated oncoproteins, playing vital roles in lipid metabolism and tumor aggressiveness with broad effects. However, it is still largely unknown about the regulating mechanisms of MYC in osteosarcoma (OS). In this study, we identify a circRNA with Reduced Expression in OS (termed as circREOS) generated from MYC gene, as a novel regulator of MYC and OS progression. CircREOS is down-regulated in OS cells and localized in the nucleus. CircREOS suppresses MYC expression, lipid metabolism and growth, invasion in OS cells. Mechanically, circREOS physically interacts with HuR (human antigen R) protein, and subsequently restrains its binding and activation on the 3'-UTR (untranslated region) of MYC mRNA, resulting in down-regulation of MYC and inhibition of OS. Moreover, circREOS serves as a tumor suppressor via targeting lipid metabolism. CircREOS reduces FASN expression and lipid accumulation through inhibiting MYC-facilitated FASN regulation. Taken together, these results indicate that circREOS suppress lipid synthesis and OS progression through inhibiting HuR-mediated MYC activation, providing a potential therapeutic target for OS.

## Introduction

Osteosarcoma (OS), usually occurring in the long diaphyseal region, is the most common primary malignant bone tumor of children and adolescents 1. Although neoadjuvant chemotherapy improves the overall efficacy of OS and improves the 5-year overall survival rate to about 60%, the 5-year survival rate is less than 30% of OS with metastasis 2, while about 10-15% of OS patients with initial visit have lung metastasis 3. As a result, it is urgent to further investigate the cellular regulatory mechanisms of tumor metastasis, and develop new therapeutic targets for OS.

MYC protein, a transcription factor encoded by the MYC proto-oncogene, is one of the most frequently activated oncoproteins playing important roles in cellular physiology of cancer 4. Accumulating evidence has indicated that aberrations or excessive expression of MYC-mediated regulation by differential mechanisms exhibit in many types of cancer, and MYC inhibition can lead to sustained tumor suppression in some preclinical models 5. Recent studies have shown that MYC triggers selective gene activation to participate in cancer metabolism reprogramming, and thus promoting cancer cell growth and invasion 6, 7. For example, MYC and sterol-regulated element-binding protein (SERBP1) could work together to activate fatty acid synthesis (FASN) and promote lipid metabolism and tumorigenesis 8. Besides, emerging studies suggest the key role of MYC in OS progression 9, however, the underlying mechanisms in OS tumorigenesis and aggressiveness about MYC regulation still remain unknown.

CircRNAs (Circular RNAs), emerged as a class of stable RNA molecules formed by non-canonical or back-splicing method into a circular structure, are described as key regulators of its host gene expression 10, 11. Notably, It is reported that circRNAs play vital roles in regulating cellular lipid metabolism 12, 13. For example, circCAPRIN1 promotes colorectal cancer lipid synthesis via interacting with STAT2 and activating ACC1 expression 14; circACC1 serves as an activator of AMPK signaling to promote fatty acid β-oxidation and glycolysis in cancer 15. Emerging studies show that circRNAs are aberrantly expressed in cancer, and are involved in the regulation of proliferation, invasion, and chemo-resistance of OS 16, 17. For example, circTADA2A promotes OS progression through enhanced CREB3 expression by miR-203a-3p sponging 18. In a previous work, we have found that circ-CTNNB1 binds with the m6A regulator of RBM15 (RNA binding motif protein 15), and facilitates RBM15-mediated m6A modification to drive aerobic glycolysis and progression of OS 19. Whereas, the expression profile and function of circRNAs in regulating OS remains largely unknown.

In this study, we identify a novel circRNA generated from MYC gene (circREOS) as a vital suppressor of MYC-mediated lipid metabolism and OS progression. CircREOS is down-expressed in OS cells, and *in vitro* experiments demonstrated that circREOS inhibited lipid synthesis and OS progression. Mechanistically, circREOS physically interacts with HuR, and subsequently restrains its binding and activation on the 3'-untranslated region of MYC, resulting in down-regulation of MYC. This led to the suppression of FASN and lipid accumulation, thus suppressing OS progression. Collectively, our results reveal the critical roles of circREOS in OS.

## Materials and methods

### Bioinformatics data mining

The R2 biologist web (R2: Genomics Analysis and Visualization Platform, http://r2.amc.nl) was used to analyze the clinical significance of MYC in OS, and the data source was GSE42352 from Gene Expression Omnibus (GEO) database. The Kaplan-Meier analysis of OS in the R2 platform used an established data set of “Mixed Osteosarcoma (Mesenchymal)-Kuijjer-127-vst-ilmnhwg6v2”. All of the high-grade osteosarcoma samples including pre-treatment biopsy (n=84) and resection (n=4) in the R2 platform were selected for gene expression analysis. The original data used for MYC gene expression analysis from R2 Platform was listed in Supplementary Table S1, S2.

### Cell culture

Human hFOB 1.19, and OS cell lines, including 143B, HOS, SJSA-1 and MG-63 were cultured with 10% fetal bovine serum (Gibco, USA).

### RT-PCR and RT-qPCR

RNA sample was extracted with Fast Pure Viral RNA Mini Kit (Vazyme). Reverse transcription was performed with the Transcriptor First Strand cDNA Synthesis Kit (Roche), and real-time PCR were executed with SYBR Green PCR Master Mix (Applied Biosystems). The primer sets were described in Supplementary Table S3. The 2^-ΔΔCt^ method was used for relative expression analysis.

### Western blot

Total protein from OS cells was extracted with RIPA lysis buffer (Thermo Fisher, USA), and incubated with antibodies of MYC (ab32072), HuR (ab200342), FASN (ab128870), and β-actin (ab179467; Abcam Inc., Cambridge, MA) overnight at 4 °C. The secondary antibodies were used for further incubation. Exposed membranes were scanned with the gel imaging system (Bio-Rad).

### Gene overexpression and knockdown

Linear circREOS (1811 bp) was synthesized by TSINGKE (Wuhan, China) and inserted into pLCDH-ciR (Geenseed Biotech Co., Guangzhou, China). Human MYC and HuR cDNA were purchased from Miao Ling Plasmid (Wuhan, China) and subcloned into pCMV-3Tag-1A. The insertion of Oligonucleotides (Supplementary Table S4) into GV298 (GeneChem Co., Ltd) was used for sh-circREOS construction. Stable cell lines treated with lentiviruses were obtained by selection with puromycin (2 μg/mL) for 2-3 weeks.

### Actinomycin D experiments

After 48h of stable cell growth, the OS cells were incubated with 1μg/ml actinomycin D for 0, 2, 4, 6h. After treatment, the expression levels of mRNA were detected by RT-qPCR.

### Dual-luciferase reporter assay

Human FASN promoter (1927 bp) was amplified from gDNA (genomic DNA) with the primer sets (Supplementary Table S4) and inserted into pGL3-Basic vector (Promega). MYC luciferase reporter was established by inserting three canonical MYC binding sites (Supplementary Table S4) into a modified pGL3-Basic vector with a minimal promoter. Transfection and dual-luciferase experiments were carried out following the instructions (Promega, Madison, WI).

### CCK8 (Cell Counting Kit-8) assay

The treated 143B or MG-63 cells (2000 cells/well) were transplanted onto 96-well tissue culture plates, performed with the manufacturer's instructions (CCK8, MCE), and measured with the microplate reader at 450 nm absorbance (Varioskan LUX, Thermo Fisher, USA).

### Colony formation assay

Stable transfected 143B or MG-63 cells were uniformly grown with 1000 cells per well. After a period of culture, cells were fixed with 4% paraformaldehyde, rewashed in PBS, and finally the 1% crystal violet was used for staining. The excess dye was washed away and photographed, and the number of clonal colonies was counted.

### Invasion assay

Transwell assay for the invasion capacity were carried out by seeding OS cells into the upper chamber (Corning) with serum-free medium. The experiment was conducted according to the operation procedure of Corning Matrigel glue (354234). At last, 5 random fields per well were photographed and cells were counted.

### RNA fluorescence *in situ* hybridization (RNA-FISH)

RNA-FISH was performed following the manufacturer's instructions (C10910, RiboBio). Biotin-labeled probes targeting circREOS (Supplementary Table S4) were synthesized by TSINGKE (Wuhan, China). The images were analyzed via Leica STELLARIS 5 Confocal Microscope (Leica Microsystems CMS GmbH, Germany).

### Fluorescence immunocytochemical staining

Cells were treated with antibody specific for HuR (ab200342; Abcam Inc., Cambridge, MA) at 4 °C overnight, and incubated with fluorescent secondary antibody and DAPI staining. The images were photographed under Leica STELLARIS 5 Confocal Microscope (Leica Microsystems CMS GmbH, Germany).

### Nile Red staining

Characterization of lipid synthesis was performed using Nile Red staining (72485, Sigma). The OS cells were stained following the instructions and images were photographed by the Leica STELLARIS 5 Confocal Microscope (Leica Microsystems CMS GmbH, Germany).

### RNA immunoprecipitation (RIP)

RIP assays were conducted following the instructions of Kit (Millipore, Bedford, MA, USA), with antibodies for HuR (ab200342; Abcam Inc., Cambridge, MA). Co-precipitated RNA was assessed by RT-PCR assays.

### Statistical analysis

Data were presented as the mean ± standard deviation (SD) with GraphPad Prism 9.4.0 (La Jolla, USA). Student's t-test or one-way ANOVA analysis were used in the statistical analysis. P < 0.05 was considered to indicate a statistically significant difference.

## Results

### CircREOS is down expressed in OS cells and localized in nucleus

**Figure 1:**
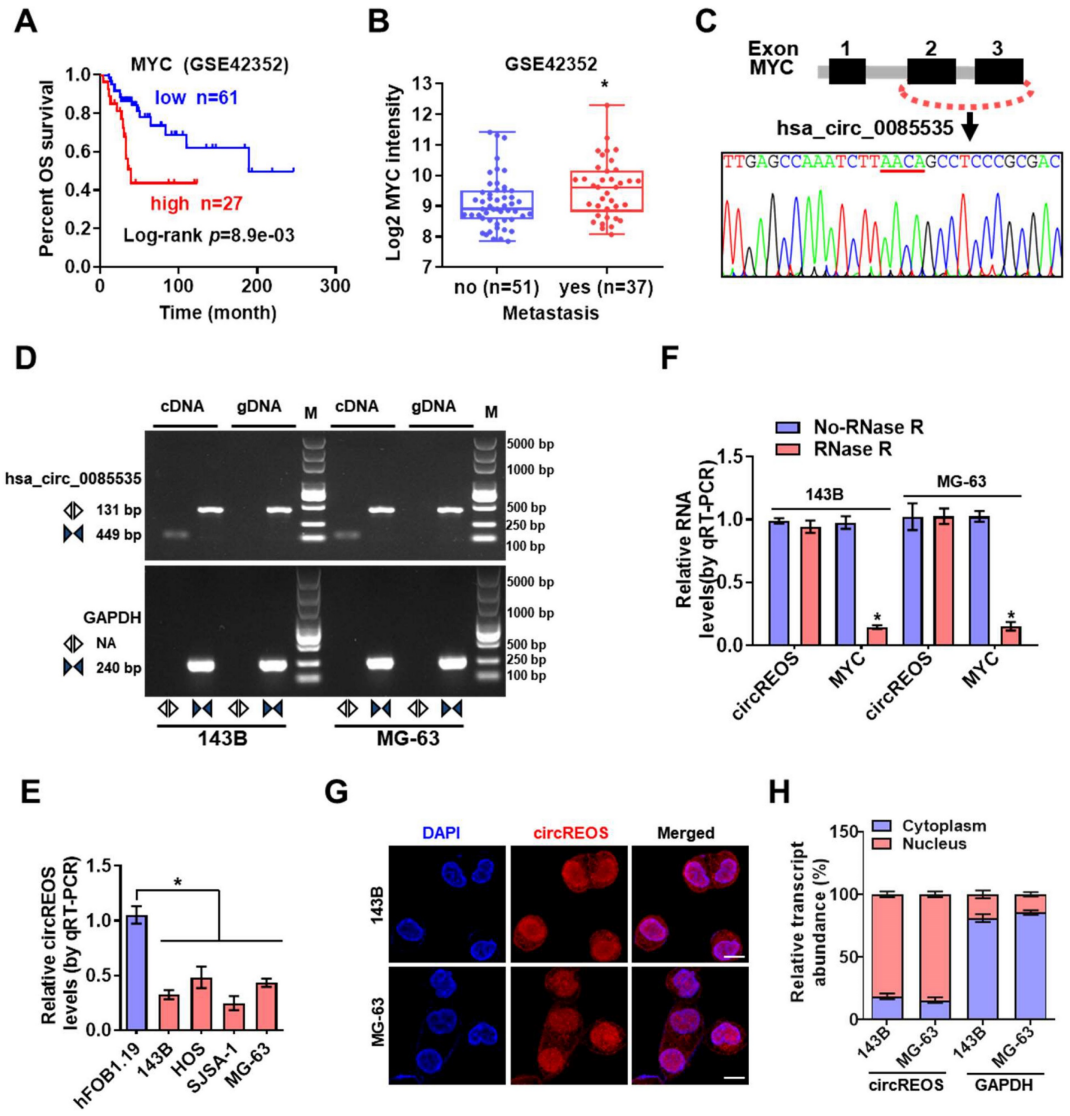
** circREOS is down regulated in OS cells and localized in nucleus. (A, B)** Kaplan-Meier and expression of MYC analyzed on R2 platform (R2: Genomics Analysis and Visualization Platform, http://r2.amc.nl) indicating the overall survival and metastasis of OS. **(C)** Hsa_circ_0085535 derived from MYC was confirmed by Sanger sequencing. **(D)** RT-PCR assay showed that the presence of circREOS with convergent and divergent primers from cDNA or gDNA of 143B and MG-63 cell lines using GAPDH as the negative control.** (E)** qRT-PCR assay showing the expression levels of circREOS in OS cells and hFOB 1.19 cells.** (F)** The expression of circREOS and MYC were analyzed by qRT-PCR after RNase R treatment in 143B or MG-63 cells.** (G, H)** RNA-FISH and qRT-PCR confirmed the localization of circREOS in 143B and MG-63 cells. Scale bar, 10 μm. *P < 0.05. Representative data from at least 3 experiments with comparable results are shown.

Through the R2 biologist web analysis with the established data set of “Mixed Osteosarcoma (Mesenchymal) -Kuijjer-127-vst-ilmnhwg6v2”, whose data source is GSE42352, we found that high expression of MYC was positively correlated with poor prognosis and enhanced metastasis in OS patients (Fig. 1A, 1B; Supplementary Table S1). Mining of the circRNA databases of circBase 20 and circInteractome 21 revealed three potential circRNAs generated from MYC gene. Further RT-PCR and Sanger sequencing validated two detectable circRNAs in OS cells (Fig. 1C, D; Supplementary Fig. S1A, B). Notably, the divergent primers of circRNA can only amplify PCR products from cDNA, not from gDNA in OS cells (Fig. 1D). We then detected these two circRNAs in OS cells, and only the endogenous level of hsa_circ_0085535, generated from exon 2 and exon 3 circularization of MYC, was consistently reduced in OS cells than that of hFOB 1.19 cells (Fig. 1E; Supplementary Fig. S1C). Therefore, this circRNA with Reduced Expression in OS, termed as circREOS, was selected for further analysis. Besides, circREOS was resistant to RNase R digestion in 143B and MG-63 cells (Fig. 1F), indicating that circRNA was more stable than the linear mRNA structure. Next, RNA fluorescence *in situ* hybridization (RNA-FISH, Fig. 1G) analysis and cytoplasmic separation experiments (Fig. 1H) confirmed that circREOS was mainly expressed in the nucleus of OS cells.

### CircREOS suppresses MYC expression and regulates lipid metabolism in OS

**Figure 2:**
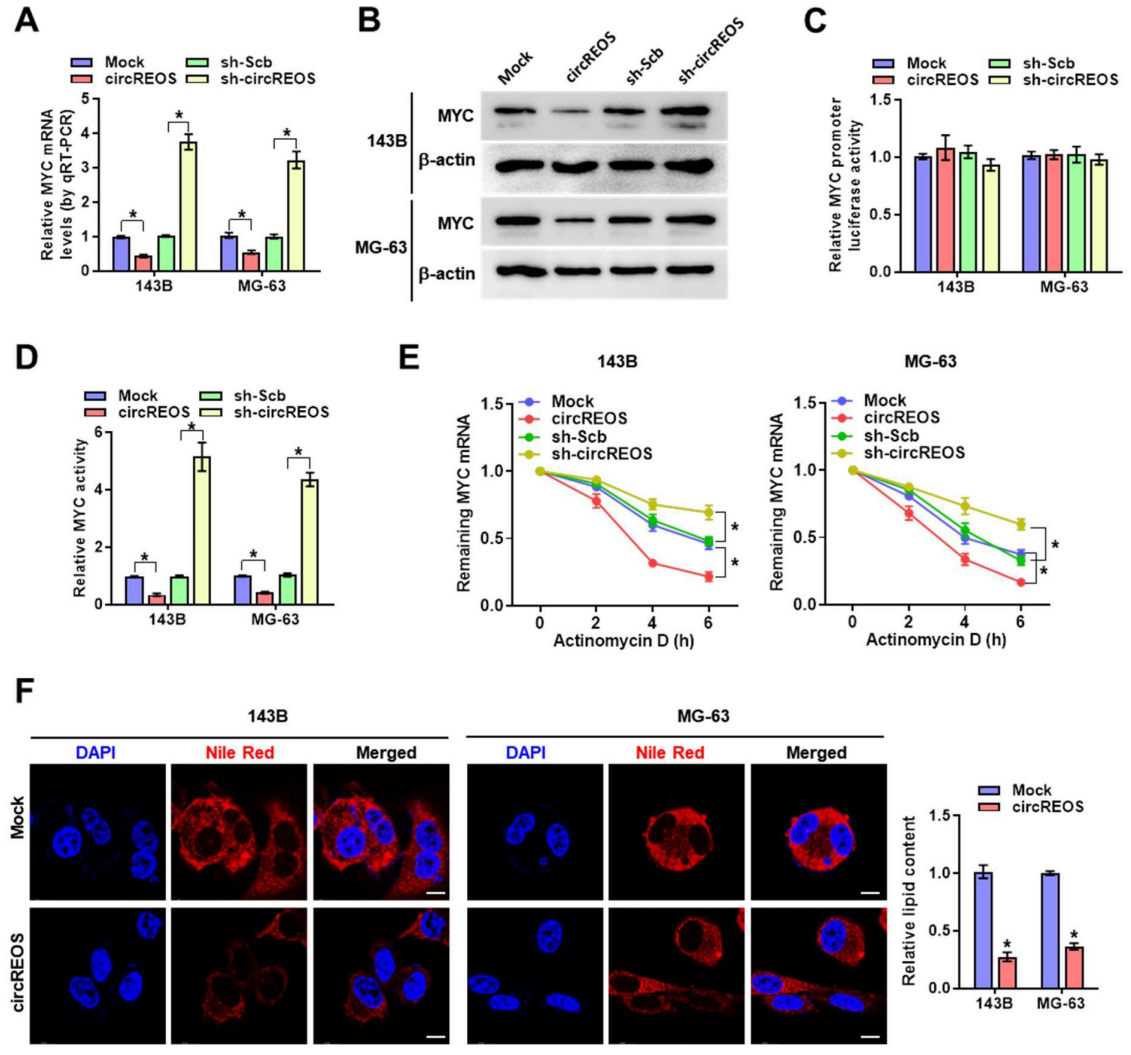
** circREOS suppresses MYC expression and regulates lipid metabolism in OS. (A-D)** Real-time qRT-PCR**(A)**, western blot **(B)** and dual-luciferase reporter **(C, D)** assay revealing the mRNA, protein, promoter activity, and binding activity of MYC in 143B and MG-63 cells stably transfected with mock, circREOS, scrambled shRNA (sh-Scb), and sh-circREOS.** (E)** qRT-PCR assay indicating the remaining MYC mRNA in the treated OS cells with Actinomycin D. **(F)** Nile Red staining assay showing the lipid synthesis in 143B and MG-63 cells stably transfected with mock and circREOS. Scale bar, 10 μm. *P < 0.05. Representative data from at least 3 experiments with comparable results are shown.

Emerging evidence has confirmed that circRNAs are key regulators of its host gene 22, 23, and then, we explored the potential cis-regulation of circREOS on MYC. Overexpression or knockdown of circREOS reduced or increased the mRNA and protein expression of MYC in 143B and MG-63 cells (Fig. 2A, B), while the promoter activity of MYC in OS cells was unaffected (Fig. 2C). In the luciferase reporter assay with three canonical MYC binding motifs (http://motifmap.ics.uci.edu/), overexpression or knockdown of circREOS decreased or promoted MYC activity in OS cells, respectively (Fig. 2D). Then we used actinomycin D, a well-established inhibitor of RNA polymerase II, to interfere with transcription and observed the half-life of mRNA. Notably, the stable ectopic expression of circREOS accelerated the RNA degradation in 143B and MG-63 cells, while knockdown of circREOS produced the reverse effects (Fig. 2E). Recent studies have reported that both circRNA and MYC participate in cancer lipid reprogramming 15, 24, and thus we suppose whether circREOS can regulate lipid metabolism through MYC inhibition in OS. The result in Nile Red staining assay showed that ectopic expression of circREOS led to decreased cellular lipid levels in 143B and MG-63 cells (Fig. 2F). These findings indicated that circREOS down-regulated MYC expression and lipid accumulation in OS cells.

### CircREOS inhibits proliferation and invasion of OS cells

**Figure 3:**
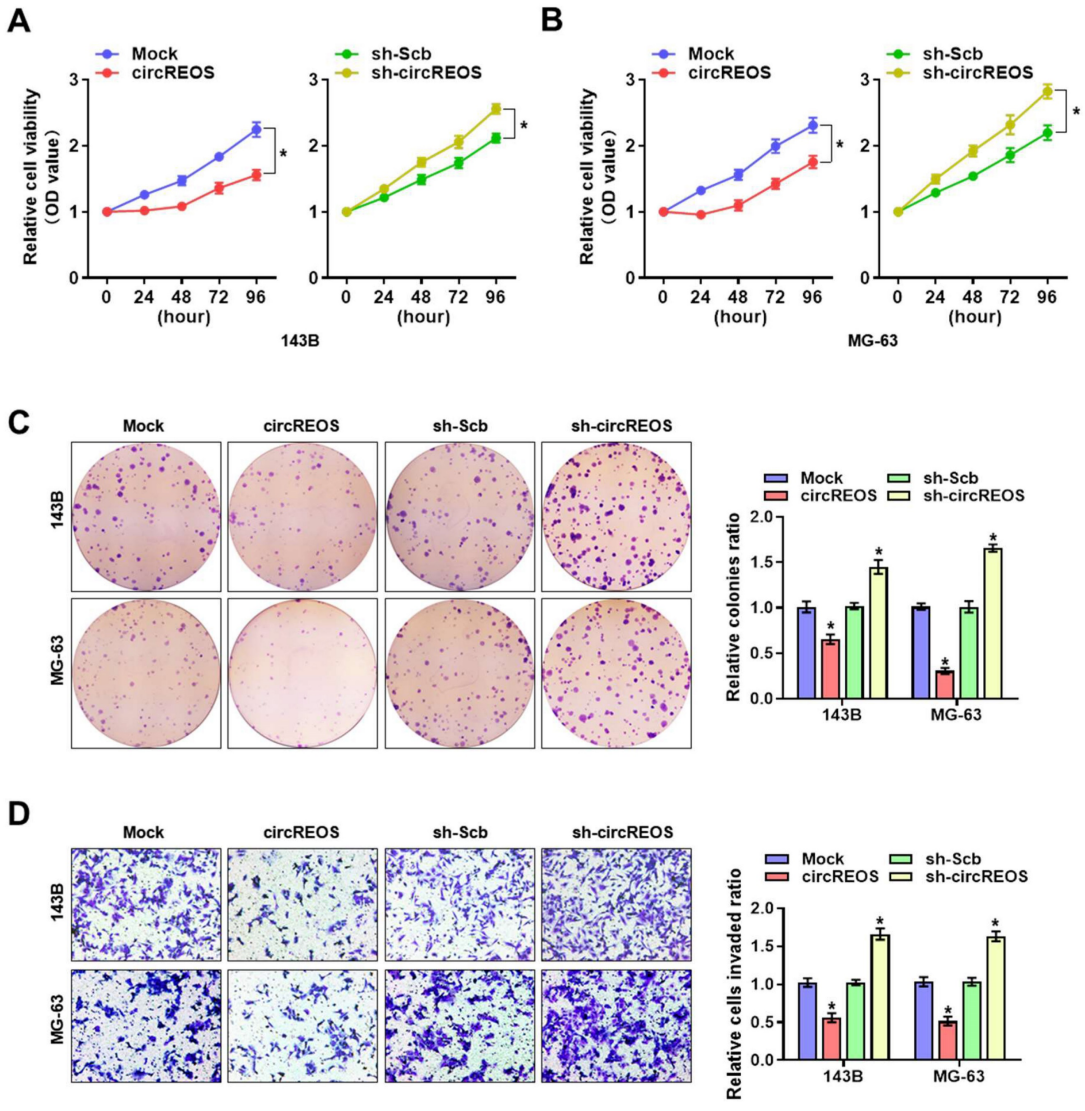
** circREOS inhibits the growth and aggressiveness of OS *in vitro*.** CCK8 **(A, B)**, colony formation **(C)**, and matrigel invasion **(D)** assays showing the *in vitro* viability, growth, and invasion capabilities of 143B and MG-63 cells stably transfected with mock, circREOS, sh-Scb, and sh-circREOS. *P < 0.05. Representative data from at least 3 experiments with comparable results are shown.

To explore the cellular function of circREOS in OS, we investigated its effect on tumorigenesis and metastasis with stable transfection of circREOS knockdown or overexpression in 143B and MG-63 cells. In order to select target for knockdown construction, three targets of siRNAs against circREOS were transfected into OS cells, and stable transfection of circREOS and shRNA against circREOS into OS cells resulted in satisfied efficiency (Supplementary Fig. S2A, B). Consistently, the results in the CCK8 (Fig. 3A, B), colony formation (Fig. 3C) and matrigel invasion assays (Fig. 3D) suggested that stable circREOS overexpression reduced the proliferation and invasion abilities of 143B and MG-63 cells, while stable circREOS knockdown produced the opposite effects. Collectively, these findings indicated that circREOS acted as an inhibitor in OS progression.

### CircREOS promotes the degradation of MYC mRNA by interacting with HuR

**Figure 4:**
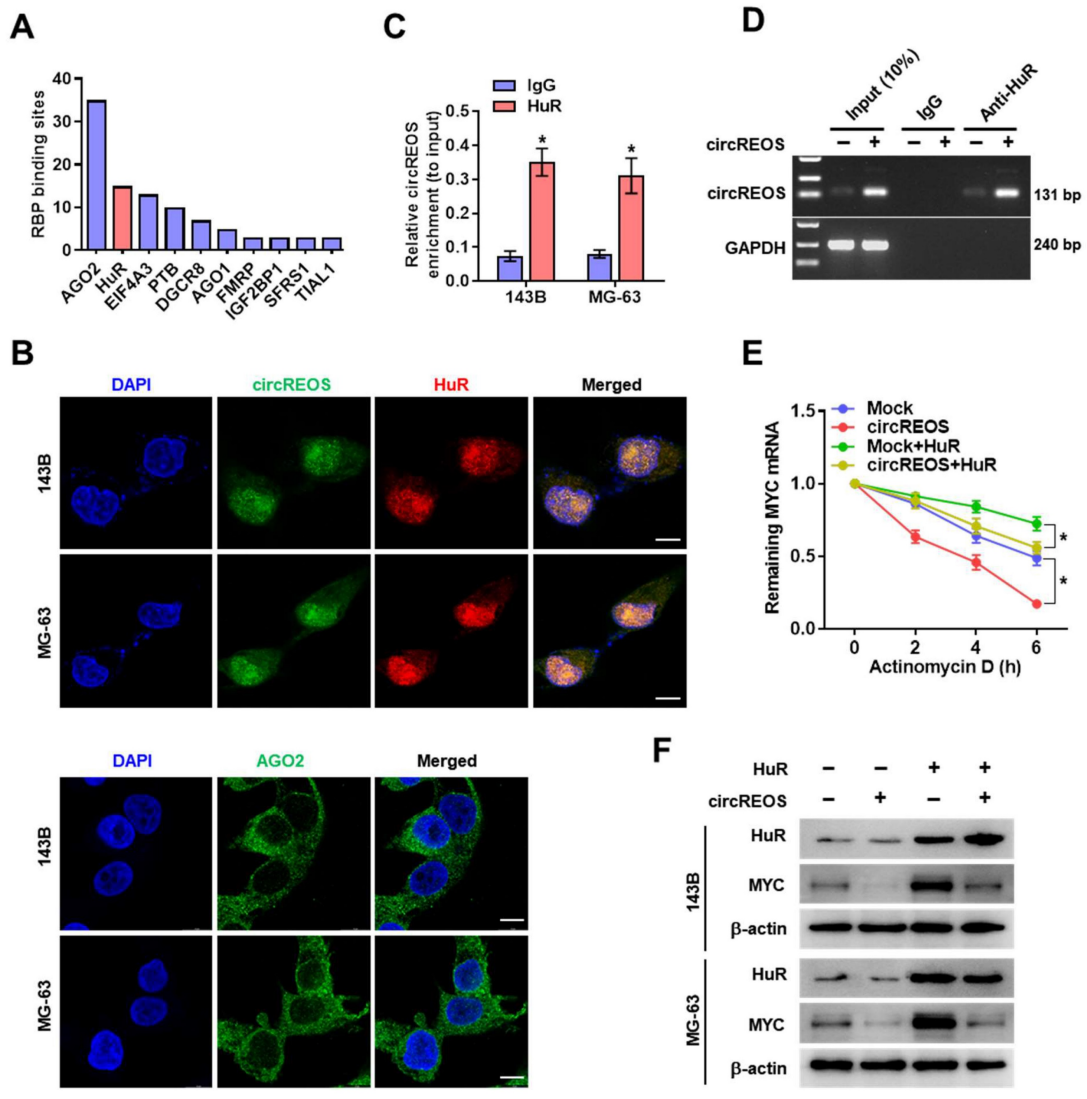
** circREOS promotes the degradation of MYC mRNA by interacting with HuR. (A)** Columnar schematic showing the top ten potential RBP of circREOS from circInteractome database.** (B)** Representative images of fluorescence showing the localization of circREOS, HuR and AGO2 in 143B and MG-63 cells. Scale bar, 10 μm. **(C)** RIP and qRT-PCR assays showing the relative circREOS enrichment (to input) of HuR in OS cells. **(D)** RIP assays showing the interaction between circREOS and HuR in 143B cells transfected with control (Mock) or circREOS.** (E)** Remaining MYC mRNA in OS cells with overexpression of HuR and circREOS was measured by actinomycin D experiments.** (F)** Western blot assays showing the protein levels of MYC and HuR in OS cells with overexpression of circREOS, and those co-transfected with HuR. *P < 0.05. Representative data from at least 3 experiments with comparable results are shown.

It is reported that circRNA regulates gene expression through interacting with RBP (RNA Binding Proteins) 25. Therefore, to explore the potential protein partner of circREOS in regulating MYC, we searched the circInteractome database to predict potential RBP, and found that AGO2 and HuR had the most possible binding sites in circREOS (Fig. 4A). Considering the main nucleus enrichment of circREOS in OS cells, we then validated its interaction with the nuclear protein of HuR, but not with the cytoplasmic protein of AGO2 in the dual RNA-FISH and IF assay (Fig. 4B). Further RIP assay confirmed the interaction of circREOS with HuR (Fig. 4C). Moreover, transfection of circREOS resulted in its enhanced binding in RNA co-precipitated complex by HuR antibody in 143B cells (Fig. 4D). Notably, HuR overexpression abolished the repression of the half-life and expression of mRNA, and protein levels of MYC induced by circREOS in 143B and MG-63 cells (Fig. 4E, F; Supplementary Fig. S3A, B). These results suggested that circREOS interacted with HuR to inhibit MYC expression.

### CircREOS regulates lipid synthesis via reducing MYC-medicated FASN activation

**Figure 5:**
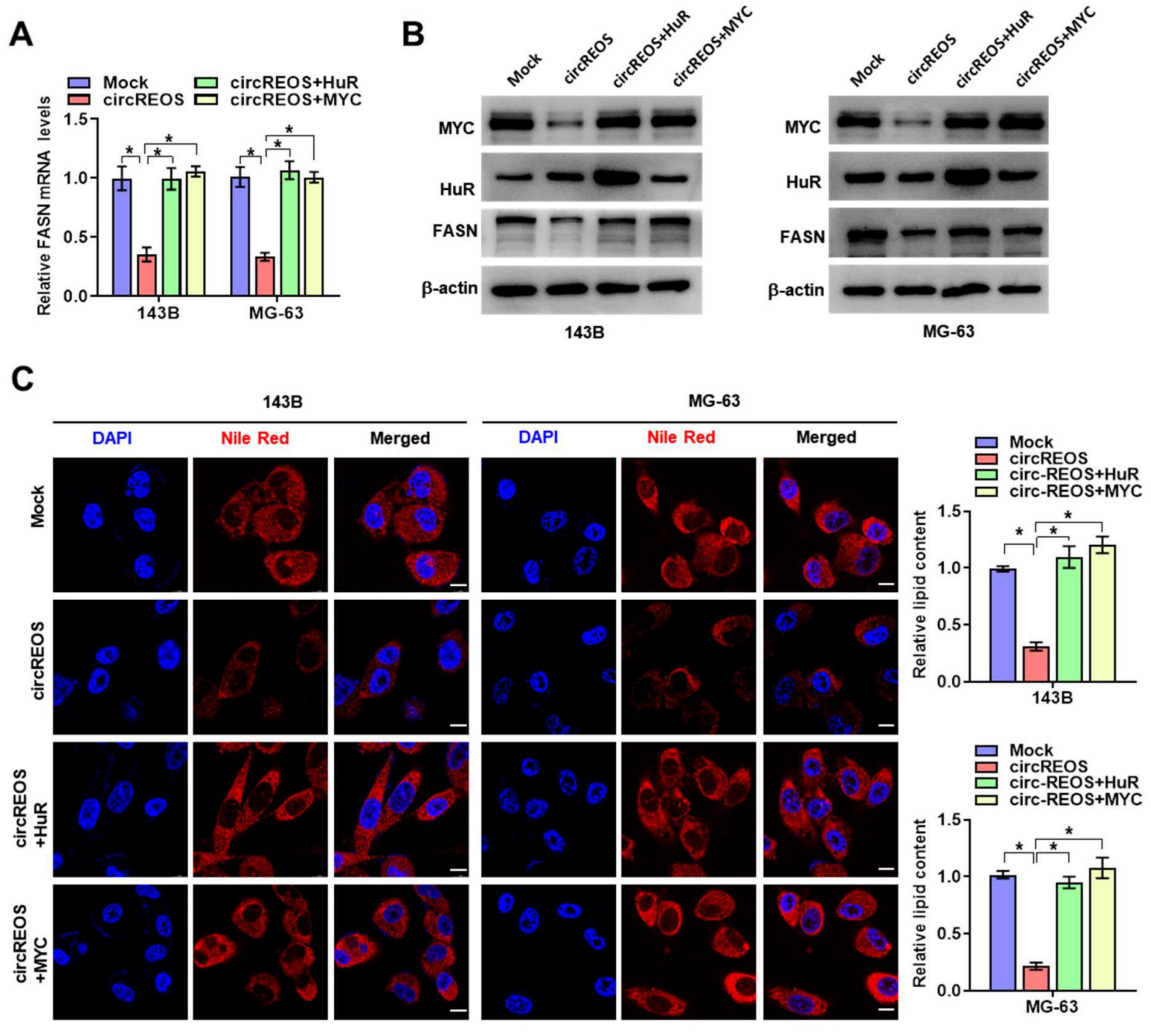
** circREOS regulated lipid synthesis via reduced MYC-medicated FASN activation**. **(A, B)** qRT-PCR (A) and western blot (B) assays revealing the transcript and protein expression levels of FASN mRNA in 143B and MG-63 cells stably transfected with circREOS or co-transfected with HuR or MYC. **(C)** Nile Red staining assay showing the lipid synthesis levels in 143B and MG-63 cells transfected with circREOS, and co-transfected with HuR or MYC. Scale bar, 10 μm. *P < 0.05. Representative data from at least 3 experiments with comparable results are shown.

Previous results have shown that circRNAs play vital roles in regulating cellular lipid metabolism in cancer 15, 26, 27, and FASN is a key regulator in cancer pathogenesis catalyzing the final steps of fatty acids biogenesis in the de novo 28. Besides, MYC is reported to occupy at the promoter of FASN to facilitate lipid metabolism and tumorigenesis 29. Therefore, we raised the hypothesis that FASN may be a target of circREOS-HuR-MYC axis in the lipid reprograming in OS. Ectopic expression of circREOS resulted in an obvious repression in the promoter activity (Supplementary Fig. S3C), mRNA (Fig. 5A), and protein (Fig. 5B) levels of FASN and lipid synthesis activity (Fig. 5C), which were abolished by ectopic expression of HuR or MYC in 143B and MG-63 cells, respectively.

### CircREOS suppresses cancer progression through MYC inhibition

**Figure 6:**
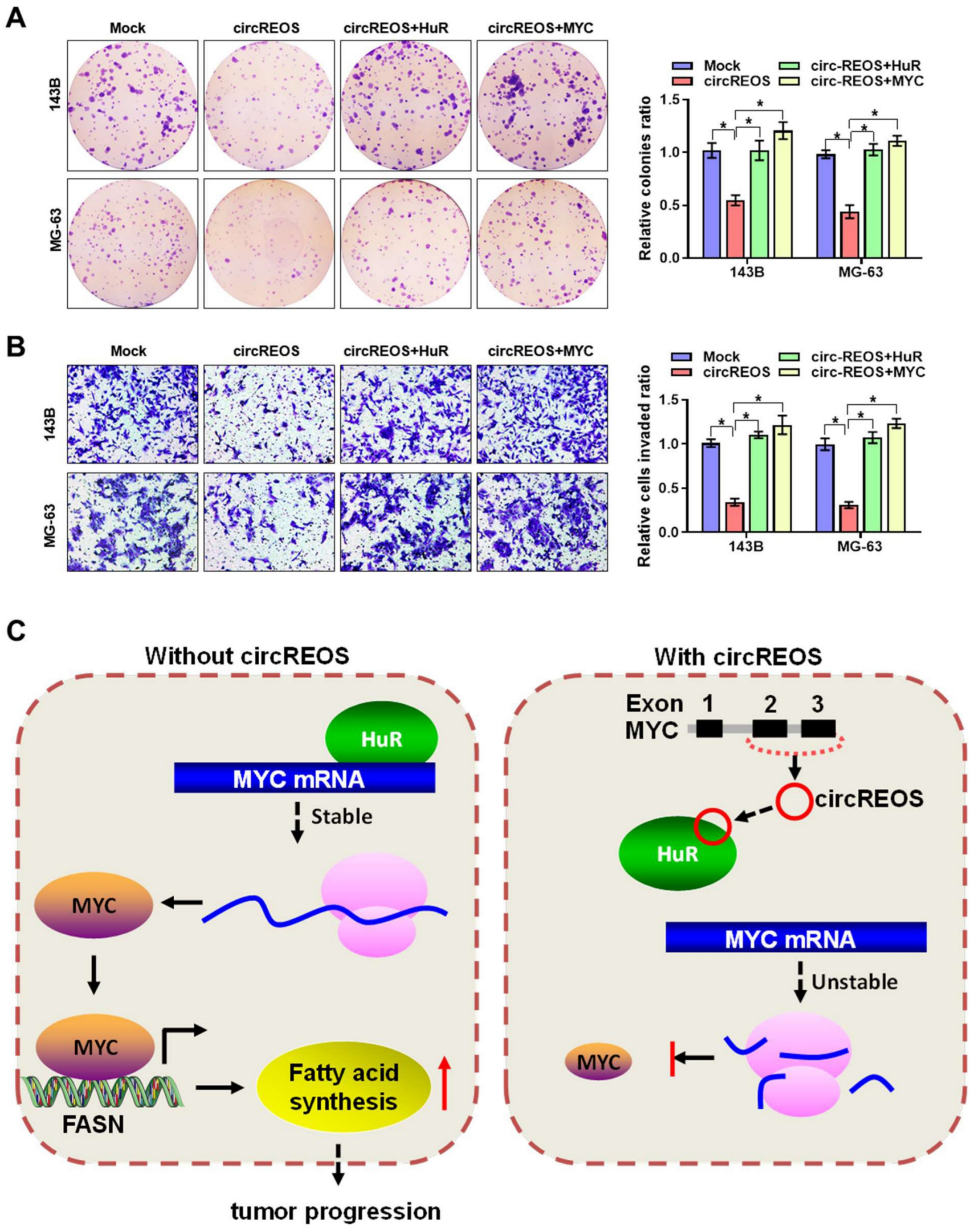
** circREOS suppresses lipid synthesis and OS progression through MYC inhibition**. **(A, B)** Colony formation **(A)**, and matrigel invasion **(B)** assays showing the growth and invasion capabilities of 143B and MG-63 cells stably transfected with circREOS, and co-transfected with HuR or MYC. **(C)** Mechanisms underlying circREOS inhibited OS progression. *P < 0.05. Representative data from at least 3 experiments with comparable results are shown.

The interaction effects between circREOS and HuR in regulating the expression of MYC and cancer progression in OS cells was further investigated. Importantly, overexpression of circREOS inhibited the growth and invasion capacity of OS cells, which can be rescued by HuR or MYC overexpression (Fig. 6A, B). Taken together, these results suggest that circREOS inhibited lipid synthesis and OS progression through inhibiting HuR-mediated MYC activation (Fig. 6C).

## Discussion

Recent studies have demonstrated that circRNAs function as important agonist of tumorigenesis or suppressors in human cancers, such as miRNA sponges, RBP binding partners, transcriptional regulators and templates for protein translation 30-32. Numerous studies have demonstrated that circRNA can control parental gene expression as a loop. For example, Exon-intron circular RNAs named circ-EIF3J is able to promote its host gene EIF3J transcription by interacting with Pol II transcription complex and U1 snRNP 33; Ectopic expression of circ-Foxo3 increased Foxo3 protein levels, and promoted stress-induced apoptosis and suppressed tumor growth 34. In this study, we discover that circREOS is a potential tumor suppressor in OS. Moreover, overexpression of circREOS suppresses the lipid synthesis, growth and aggressiveness of OS cells. Mechanistically, circREOS binds with HuR to restrain its enrichment on the 3'-UTR of MYC mRNA, leading to down-regulation of MYC and then reduced FASN expression and lipid accumulation. Taken together, these results indicate that circREOS suppress lipid synthesis and OS progression through inhibiting HuR-mediated MYC activation, providing a potential therapeutic target for OS.

MYC is a major regulatory factor of gene transcription and a powerful driver of transformation, playing an active role in biological processes by recruiting multiple protein complexes 4. Studies have revealed that MYC is elevated in OS and is associated with cell growth and aggressiveness 9, and circRNA could participate in the regulation of MYC. For example, CircECE1 binds with MYC to block its ubiquitination mediated degradation, thereby promoting the progression of OS 35; CircMYC promotes small cell lung cancer progression by miR-145/MMP2 axis 36. However, the MYC produced-circRNAs in regulating its own expression or function has not been reported yet. In this study, we identified a novel circRNA (circREOS) produced from exon 2 and exon 3 circularization of MYC gene regulates the expression of MYC itself as negative feedback. Besides, emerging studies have suggested the compelling function of MYC in cancer lipid metabolism. For example, ectopic expression of MYC in prostate cancer leads to an increase in the expression of ACLY, ACC1, and FASN, resulting in promoted lipid accumulation 37. In addition, in MYC-induced hepatocellular carcinoma, FASN suppressed by gene silencing or soluble inhibitors inhibited proliferation and induced apoptosis 38. As a central regulatory factor of lipid metabolism, FASN plays a key role in the growth and survival of lipid-derived tumors 39-41.

HuR is reported to regulate the half-life and steady state levels of the corresponding mRNA through binding with the AU-rich elements of mRNA 42. It is indeed one of the most studied RBP shuttling between the nuclear and the cytoplasm, and the enrichment outside the nuclear appears to be intimately associated with the mRNA-stabilizing regulation 43. Since HuR is necessary for the activation of many oncogenes, including MYC 44, down-regulation in HuR protein levels or inhibition of HuR activity are with significant value, suggesting HuR as a promising therapeutic target in cancer. In the current study, we identify that circREOS can competitively bind with HuR to inhibit HuR-mediated target gene activation. Specifically, circREOS blocks the interaction between HuR and MYC mRNA, resulting in a significant downregulation of MYC in OS, suggesting a potential therapeutics target of OS.

## Conclusions

Our study demonstrates a novel circular RNA, termed as circREOS, is down-expressed in OS cells and predominantly in the nucleus. CircREOS can act as a sponge of protein through interacting with HuR to suppress its binding activity, and suppress the lipid metabolism, proliferation and invasion of OS cells *in vitro*. In summary, our study reveals that circREOS inhibits HuR-mediated MYC activation and thus repressed FASN expression, lipid metabolism and OS progression. Therefore, this finding illustrates the vital role of circREOS/HuR/MYC/FASN axis in OS progression.

## Supplementary Material

Supplementary figures and tables.
