# Supplementary material for: CircREOS suppresses lipid synthesis and osteosarcoma progression through inhibiting HuR-mediated MYC activation
Source: J Cancer. 2023 Apr 2;14(6):916–26. doi: 10.7150/jca.83106 (PMC10158517; doi:10.7150/jca.83106)
Supplement: Supplementary file 1 — Supplementary figures and tables. [file jcav14p0916s1.pdf]

Supplementary Table

Table S1

The clinical relevance of MYC in OS from R2 platform

Characteristic	Low expression of MYC	High expression of MYC	p
	**n=61**	**n=27**	
**Sex**			0.187
Female	24 (27.3%)	6 (6.8%)	
Male	37 (42%)	21 (23.9%)	
**Death**			0.107
No	46 (52.3%)	15 (17%)	
Yes	15 (17%)	12 (13.6%)	
**Metastasis**			**0.004** *
M0	42 (47.7%)	9 (10.2%)	
M1	19 (21.6%)	18 (20.5%)	
**Histsubtype**			**< 0.001** *
chondroblastic	1 (1.1%)	8 (9.1%)	
fibroblastic	9 (10.2%)	0 (0%)	
osteoblastic	38 (43.2%)	17 (19.3%)	
other	9 (10.2%)	2 (2.3%)	
telangiectatic	4 (4.5%)	0 (0%)	
**Huvos**			0.683
1	12 (15%)	6 (7.5%)	
2	15 (18.8%)	9 (11.2%)	
3	17 (21.2%)	9 (11.2%)	
4	10 (12.5%)	2 (2.5%)	
**Location**			0.370
femur	32 (36.4%)	12 (13.6%)	
humerus	8 (9.1%)	3 (3.4%)	
other	4 (4.5%)	0 (0%)	
tibia/fibula	17 (19.3%)	12 (13.6%)	
**Site**			1.000
left	27 (42.2%)	13 (20.3%)	
right	17 (26.6%)	7 (10.9%)	
**Age (m), median**	192 (160, 261)	198 (179, 220.5)	0.946
**Survival time,**	45.5 (31.53, 79.25)	31 (23, 38)	**0.003** *

*P<0.05 indicates a significant relationship among the variables

Table S2 The original data analyzed from the R2 Platform

Sample name	MYC expression	Deceased	metastasis	Time (m)	Histsubtype	Huvos	Location	Sex	Site	Type	Age (m)
CLET_L1016	9.867433196	TRUE	TRUE	10	osteoblastic	1	tibia/fibula	m	left	biopsy	200
CLET_L1085	9.816983623	TRUE	TRUE	39	chondroblastic	3	tibia/fibula	m	left	biopsy	223
CLET_L1368	9.064203842	FALSE	TRUE	95	osteoblastic	4	femur	m	left	biopsy	177
CLET_L1369	8.270061673	TRUE	TRUE	83	osteoblastic	2	femur	f	right	biopsy	212
CLET_L1370	8.53954658	FALSE	FALSE	246	osteoblastic	3	femur	m	left	biopsy	216
CLET_L1372	9.610840298	TRUE	TRUE	25	telangiectatic	1	femur	m	right	biopsy	200
CLET_L1376	8.411510988	TRUE	TRUE	40	telangiectatic	1	tibia/fibula	f	right	biopsy	164
CLET_L1378	9.071194179	TRUE	FALSE	14	broblastic	2	femur	f	left	biopsy	197
CLET_L1382	8.820178962	FALSE	FALSE	143	osteoblastic	2	tibia/fibula	m	right	biopsy	175
CLET_L1385	9.236253425	TRUE	TRUE	11	osteoblastic	nd	humerus	m	left	biopsy	200
CLET_L1386	9.50442177	FALSE	FALSE	105	osteoblastic	1	femur	m	left	biopsy	101
CLET_L2068	8.412358137	FALSE	FALSE	39.86666667	osteoblastic	3	femur	m	right	biopsy	149.75342
CLET_L2178	8.900866808	FALSE	FALSE	36.83333333	osteoblastic	3	femur	f	right	biopsy	165.20548
CLET_L2281	8.994353437	TRUE	TRUE	64	osteoblastic	2	tibia/fibula	m	left	resection	212
CLET_L2289	8.5360529	FALSE	FALSE	135	fibroblastic	2	femur	m	left	resection	136
CLET_L2290	8.913787	TRUE	TRUE	13	fibroblastic	2	femur	m	left	resection	440
CLET_L2292	8.602142091	FALSE	FALSE	78	osteoblastic	3	tibia/fibula	m	left	biopsy	136
CLET_L2294	8.556122818	FALSE	FALSE	147	osteoblastic	1	femur	f	left	resection	205
CLET_L2295	8.99095486	FALSE	FALSE	97	anaplastic	2	femur	f	left	biopsy	487
CLET_L2296	9.881572857	TRUE	TRUE	33	osteoblastic	3	tibia/fibula	m	left	biopsy	198
CLET_L2297	8.720757277	FALSE	FALSE	77	osteoblastic	2	tibia/fibula	f	left	biopsy	137
CLET_L2301	8.519243094	TRUE	TRUE	47	osteoblastic	2	tibia/fibula	m	left	biopsy	304
CLET_L2302	9.73809226	FALSE	FALSE	120	chondroblastic	2	humerus	f	right	biopsy	229
CLET_L2347	9.367414751	FALSE	FALSE	32.6	telangiectatic	3	femur	f	right	biopsy	164.97534
CLET_L2376	9.136991112	FALSE	FALSE	30.13333333	osteoblastic	3	humerus	f	left	biopsy	113.72055
CLET_L2611	9.269360484	FALSE	FALSE	91	osteoblastic	3	femur	f	right	biopsy	242
CLET_L2613	10.21164499	FALSE	FALSE	40	osteoblastic	3	tibia/fibula	m	right	biopsy	181
CLET_L2614	10.83928254	TRUE	TRUE	29	osteoblastic	2	femur	m	left	biopsy	165
CLET_L2615	9.196233261	FALSE	FALSE	35	osteoblastic	3	tibia/fibula	m	left	biopsy	261
CLET_L2616	10.25632665	FALSE	TRUE	32	osteoblastic	2	tibia/fibula	m	left	biopsy	218
CLET_L2617	9.249587556	FALSE	FALSE	32	osteoblastic	4	tibia/fibula	f	left	biopsy	181
CLET_L2618	10.80275868	FALSE	TRUE	31	osteoblastic	2	femur	m	left	biopsy	96
CLET_L2619	8.320574934	TRUE	TRUE	25	osteoblastic	1	femur	f	right	biopsy	128
CLET_L2620	9.613605318	FALSE	FALSE	219	osteoblastic	2	femur	m	left	biopsy	217
CLET_L3431	9.741635516	TRUE	TRUE	11	osteoblastic	1	humerus	f	nd	biopsy	81
CLET_L3432	10.80429239	FALSE	TRUE	37	chondroblastic	2	tibia/fibula	m	nd	biopsy	144
CLET_L3433	9.821773982	TRUE	TRUE	33	osteoblastic	1	femur	f	left	biopsy	204
CLET_L3434	9.660709039	FALSE	TRUE	25	osteoblastic	1	tibia/fibula	m	right	biopsy	181
CLET_L3435	9.847213982	TRUE	TRUE	30	osteoblastic	1	femur	m	right	biopsy	200
CLET_L3436	10.18462744	TRUE	TRUE	35	chondroblastic	3	femur	m	left	biopsy	205
CLET_L3437	10.1382718	TRUE	TRUE	27	osteoblastic	2	femur	m	nd	biopsy	183
CLET_L3438	9.418274294	FALSE	TRUE	26	osteoblastic	4	tibia/fibula	m	right	biopsy	220
CLET_L3439	9.605849867	TRUE	TRUE	18	dromyxoid	1	humerus	m	right	biopsy	385
CLET_L3440	9.860311055	FALSE	TRUE	36	osteoblastic	1	femur	m	nd	biopsy	264
CLET_L3441	10.66604643	FALSE	TRUE	123	osteoblastic	3	femur	m	nd	biopsy	228
CLET_L3442	8.594697827	TRUE	TRUE	110	osteoblastic	3	femur	f	nd	biopsy	144
CLET_L3443	8.170926525	FALSE	FALSE	63	fibroblastic	2	humerus	f	nd	biopsy	204
CLET_L3444	7.917073663	FALSE	FALSE	60	fibroblastic	1	humerus	m	nd	biopsy	264
CLET_L3445	8.278449458	FALSE	FALSE	60	telangiectatic	nd	tibia/fibula	f	nd	biopsy	696
CLET_L3446	9.041385369	TRUE	TRUE	27	giant cell rich	4	femur	m	left	biopsy	174
CLET_L3447	11.2244841	TRUE	TRUE	21	osteoblastic	2	humerus	m	left	biopsy	162
CLET_L3448	8.689299161	FALSE	TRUE	46	osteoblastic	3	femur	m	left	biopsy	170
CLET_L3449	8.999154423	FALSE	TRUE	28	fibroblastic	1	femur	f	left	biopsy	133
CLET_L3453	9.136991112	FALSE	FALSE	34	osteoblastic	4	femur	m	left	biopsy	173
CLET_L3454	8.105384749	FALSE	FALSE	57	osteoblastic	1	os ilium	m	nd	biopsy	492
CLET_L3455	10.55564363	FALSE	FALSE	18	chondroblastic	3	tibia/fibula	m	left	biopsy	237
CLET_L3456	9.00337736	FALSE	FALSE	44	fibroblastic	4	tibia/fibula	m	right	biopsy	196
CLET_L3457	8.697315122	FALSE	FALSE	33	osteoblastic	2	humerus	f	nd	biopsy	115
CLET_L3458	8.14974712	FALSE	FALSE	26	osteoblastic	3	femur	m	nd	biopsy	220
CLET_L3459	11.22857822	FALSE	FALSE	45	osteoblastic	4	tibia/fibula	f	left	biopsy	180
CLET_L3460	10.49055008	TRUE	TRUE	13	chondroblastic	2	tibia/fibula	m	right	biopsy	180
CLET_L3461	8.888438851	FALSE	FALSE	42	osteoblastic	3	femur	m	left	biopsy	184
CLET_L3462	9.73403214	FALSE	FALSE	27	osteoblastic	3	tibia/fibula	m	left	biopsy	189
CLET_L3463	8.619853147	TRUE	TRUE	18	osteoblastic	nd	humerus	m	nd	biopsy	948
CLET_L3464	9.185866545	FALSE	FALSE	28	osteoblastic	3	tibia/fibula	m	left	biopsy	144
CLET_L3465	12.28768353	TRUE	TRUE	4	chondroblastic	nd	femur	f	left	biopsy	129
CLET_L3466	8.826230678	FALSE	FALSE	42	osteoblastic	4	femur	f	left	biopsy	189
CLET_L3467	9.515108449	FALSE	FALSE	18	osteoblastic	1	femur	m	left	biopsy	168
CLET_L3468	8.698704667	FALSE	FALSE	nd	chondroblastic	nd	pelvis	m	nd	biopsy	744
CLET_L3469	11.41235814	FALSE	FALSE	11	chondroblastic	4	femur	m	nd	biopsy	284
CLET_L3471	9.449148645	TRUE	FALSE	50	osteoblastic	nd	axial	f	nd	biopsy	300
CLET_L3472	8.057991723	FALSE	FALSE	38	osteoblastic	2	femur	m	nd	biopsy	516
CLET_L3473	8.669593751	FALSE	FALSE	48	osteoblastic	3	tibia/fibula	m	nd	biopsy	312
CLET_L3474	7.846117173	FALSE	FALSE	48	osteoblastic	1	humerus	m	nd	biopsy	192
CLET_L3475	7.917073663	FALSE	FALSE	60	fibroblastic	3	femur	m	nd	biopsy	336
CLET_L3476	8.21916852	FALSE	FALSE	41	fibroblastic	4	tibia/fibula	m	nd	biopsy	324
CLET_L3477	8.899356923	FALSE	FALSE	60	osteoblastic	nd	femur	m	nd	biopsy	192
CLET_L3533	8.777090896	FALSE	FALSE	15	oblastic giant	1	femur	m	right	biopsy	150
CLET_L3534	9.179660328	FALSE	TRUE	45	sclerosing	3	femur	f	left	biopsy	180
CLET_L3535	8.860155835	FALSE	FALSE	49	giant cell rich	2	tibia/fibula	m	right	biopsy	160
CLET_L3536	11.31373335	FALSE	FALSE	30	sclerosing	2	femur	m	nd	biopsy	178
CLET_L3537	8.070925945	TRUE	TRUE	189	fibroblastic	nd	nd	f	nd	biopsy	468
CLET_L3538	8.472487771	FALSE	TRUE	45	nd	3	femur	m	right	biopsy	102.90411
CLET_L428	9.508190931	FALSE	FALSE	193	osteoblastic	2	tibia/fibula	f	right	biopsy	192
CLET_L432	8.735386783	FALSE	FALSE	184	pleomorphic	4	femur	f	right	biopsy	111
CLET_L436	9.282393469	FALSE	FALSE	194	osteoblastic	4	tibia/fibula	f	left	biopsy	37
CLET_L975	10.15810472	FALSE	FALSE	94	anaplastic	3	femur	f	right	biopsy	244
CLET_L997	10.10118782	FALSE	FALSE	87	osteoblastic	3	tibia/fibula	m	right	biopsy	217

Table S3

Primer sets used for qPCR, RT-PCR, and RIP

Primer set 1	Primers	Sequence	Product size (bp)
ACTB	Forward	5'-TGCCCATCTACGAGGGGTATG-3'	156
	Reverse	5'-TCTCCTTAATGTCACGCACGATTT-3'	
GAPDH	Forward	5'-GCCAAGGCTGTGGGCAAGGT-3’	240
(Convergent)	Reverse	5'-GGAGGAGTGGGTGTCGCTGT-3’	
GAPDH	Forward	5'- ACAGCGACACCCACTCCTCC-3’	NA
(Divergent)	Reverse	5'- ACCTTGCCCACAGCCTTGGC-3’	
hsa_circ_0085533	Forward	5'-GTCCCTGGCTCCCCTCCTGCCT-3’	423
(Convergent)	Reverse	5'-GGAAGTGTCCCCAAATGGGC-3’	
hsa_circ_0085533	Forward	5'-GCCCATTTGGGGACACTTCC-3’	174
(Divergent)	Reverse	5'-AGGCAGGAGGGGAGCCAGGGAC-3’	
circREOS	Forward	5'-CTGCGACGAGGAGGAGAACTT-3’	449
(Convergent)	Reverse	5'-AGGTGGAGCAGACGCTGTGG-3’	
circREOS	Forward	5'-TATCATTGAGCCAAATCTTA-3’	131
(Divergent)	Reverse	5'-TAGAAGTTCTCCTCCTCGTC-3’	
hsa_circ_0085534	Forward	5'-AAACTGCCTCAAATTGGACT-3'	490
(Divergent)	Reverse	5'-AAAACCGGCTTTTATACTCA-3'	
MYC	Forward	5'-TGGTCTTCCCCTACCCTCTCA-3'	205
	Reverse	5'-TCTTCCTCATCTTCTTGTTCC-3'	
FASN	Forward	5'-CCGAGACACTCGTGGGCTA-3'	209
	Reverse	5'-CTTCAGCAGGACATTGATGCC-3'	

Table S4

Oligonucleotide sets used for constructs, siRNAs or probe

**Oligo Set**	**Sequences**
sh-Scb	5'-AGGGATACAAGCATATACCACTCGAGTGGTATATGCTTGTATCCCTC-3' (sense);
	5'-GAGGGATACAAGCATATACCACTCGAGTGGTATATGCTTGTATCCCT-3' (antisense)
sh-circREOS	5'-CCGGTTGAGCCAAATCTTAACAGCCTCTCGAGAGGCTGTTAAGATTTGGCTCATTTTTG -3' (sense);
	5’-GATCCAAAAATGAGCCAAATCTTAACAGCCTCTCGAGAGGCTGTTAAGATTTGGCTCAA-3’ (antisense)
si-circREOS #1 (target)	5’- AATCTTAACAGCCTCCCGCGA-3’;
si-circREOS #2 (target)	5’-TGAGCCAAATCTTAACAGCCT-3’
si-circREOS #3 (target)	5’-AGCCAAATCTTAACAGCCTCC-3’
pGL3-MYC Reporter	5’-CAACCACGTGCTCATAGGGCACGTGGTCTATGACCACGTGCCCA -3’ (sense)
pGL3-MYC Reporter	5’-AGCTTGGGCACGTGGTCATAGACCACGTGCCCTATGAGCACGTGGTTGGTAC -3’ (antisense);
pGL3-FASN (-1557/+370)	5’-CGGGGTACCACCTGGGAGGTTTGGAGCATGGAG-3’ (sense)
	5’-CGGAAGATCTGGAAGCGAAGGCGGCTGTTGGT-3’ (antisense)
circREOS probe sense	5’-AATCTTAACAGCCTCCCGCGA-3’;
circREOS probe antisense	5’- TCGCGGGAGGCTGTTAAGATT-3’

Supplementary Figure

Figure S1

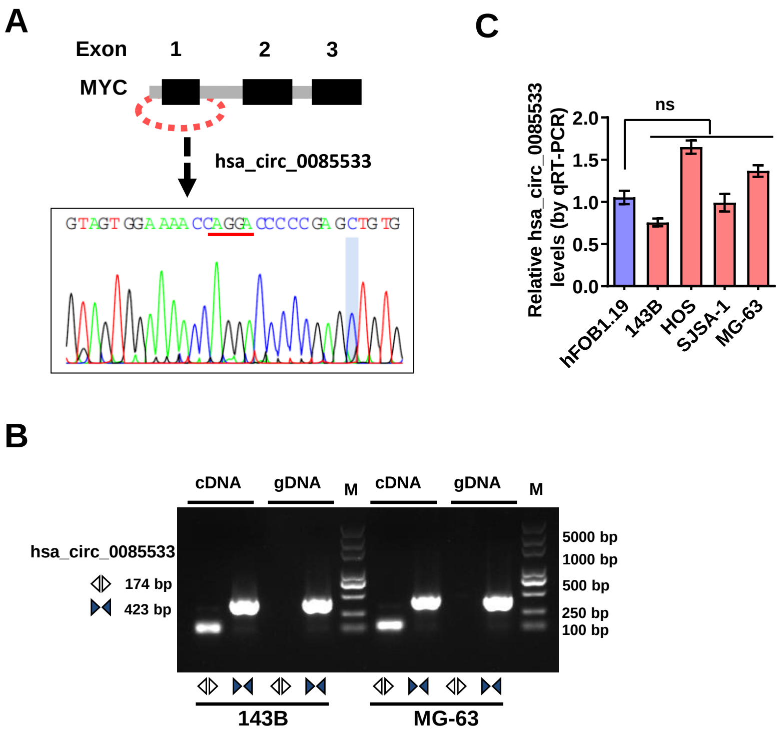

Figure S1 Expression profiles of hsa_circ_0085533. (A) Hsa_circ_0085533 derived from MYC was confirmed by Sanger sequencing. **(B)** RT-PCR assay showed that the presence of hsa_circ_0085533 with convergent and divergent primers from cDNA or gDNA of 143B and MG-63 cell lines. **(C)** The expression of hsa_circ_0085533 was analyzed by qRT-PCR in hFOB 1.19 and OS cells. ns, P$>$0.05. Representative data from at least 3 experiments with comparable results are shown.

Figure S2

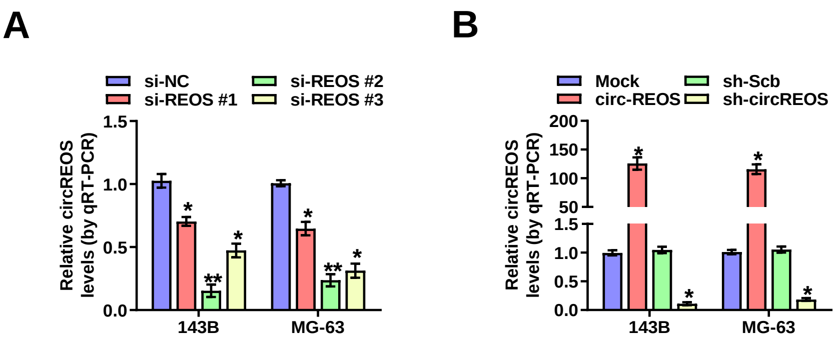

Figure S2 circREOS knockdown and overexpression in OS. (A) Real-time qRT-PCR assay showing the knockdown efficiency of the three siRNAs targeting circREOS in 143B and MG-63 cells. **(B)** Real-time qRT-PCR analysis verified the effective expression in OS cells stably transfected with circREOS overexpression or knockdown. *$P < 0.05$. Representative data from at least 3 experiments with comparable results are shown.

Figure S3

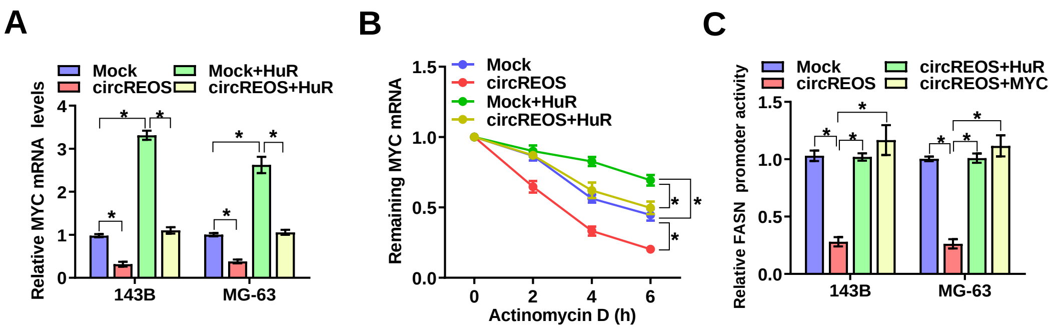

Figure S3 circREOS suppresses MYC and FASN expression in OS. (A, B) Real-time qRT-PCR assay showing the expression levels **(A)** and half-life **(B)** of MYC mRNA in OS cells stably transfected with circREOS, and those co-transfected with HuR. **(C)** Dual-luciferase reporter assay showing the promoter activity of FASN in OS cells stably transfected with circREOS, and those co-transfected with HuR or MYC. *$P < 0.05$. Representative data from at least 3 experiments with comparable results are shown.
